# Thermal adaptation of *Lactiplantibacillus plantarum* by laboratory evolution does not enhance tagatose biosynthesis

**DOI:** 10.1093/lambio/ovag084

**Published:** 2026-09-26

**Authors:** Vikas D Trivedi, Todd C Chappell, Nikhil U Nair

**Affiliations:** Department of Chemical & Biological Engineering, Tufts University, Medford, MA 02155, United States; Department of Chemical & Biological Engineering, Tufts University, Medford, MA 02155, United States; Department of Chemical & Biological Engineering, Tufts University, Medford, MA 02155, United States

**Keywords:** Heat treatment, thermal stress, LAI, LAB, lactic acid bacteria, probiotic

## Abstract

D-Tagatose is recognized as a promising sugar alternative, but its biosynthesis is limited by poor thermodynamics of isomerization from D-galactose. Higher reaction temperatures favor tagatose production, but microbial whole-cell conversion is limited by the viability of cells at elevated temperatures. This work attempts to address this by engineering *Lactiplantibacillus plantarum* for growth at elevated temperatures towards development of a high-temperature galactose-to-tagatose isomerization biocatalytic system. Using adaptive laboratory evolution (ALE), a strain capable of growth at 43°C was isolated over 85 passages of increasing thermal stress. The adaptation required using a cycling approach, where cultures were cultured at permissive (37°C) temperatures between periods of growth at elevated temperatures. At passage #85, the cells were able to grow at 43°C over multiple passages, indicating complete adaptation. They were also able to grow well at 37°C. Unfortunately, the galactose-to-tagatose conversion reaction at 37, 50, and 60°C using an L-arabinose isomerase-transformed strain revealed no benefit of using the evolved strain over the parental strain. Nevertheless, the ALE protocol and isolated strain may be of benefit for other applications.

Impact statement A food-safe microbe capable of robust growth at high temperatures may be beneficial for biocatalysis. Using adaptive laboratory evolution (ALE), this study obtained strains of *Lactiplantibacillus plantarum* WCFS1 capable of growth at 43°C (the parental strain cannot grow at 40°C). The ALE protocol required cycling of cells to lower temperatures to overcome evolutionary dead-ends. The evolved strain was used to catalyze isomerization of D-galactose to D-tagatose at elevated temperatures but did not demonstrate enhanced conversion. Despite this, the strain may be used in other applications, and future studies may also reveal how it adapted to chronic thermal stress.

## Introduction

D-Tagatose is a rare sugar with sweetness similar to sucrose but lower caloric value. Recent studies also indicate that it is anti-hyperglycemic (Rhimi et al. 2015) and prebiotic (Hans Bertelsen, Hans Andersen 2001). While tagatose is a natural sugar, it is present in very small quantities in fruits and vegetables, making extraction-based manufacturing commercially unattractive. Resultantly, there has been significant interest in using biocatalysts to synthesize tagatose from more abundant and/or inexpensive sugars. Described approaches generally fall into five categories: (i) direct isomerization of D-galactose to tagatose using L-arabinose isomerase (LAI) (Oh et al. 2006a,b, Roh et al. 2000, Kim et al. 2001, 2002, Jørgensen et al. 2004, Kim and Oh 2005, Manjasetty and Chance 2006, Zhang et al. 2007, Hong et al. 2007, Rhimi et al. 2009, 2010, Cheng et al. 2010, Xu et al. 2011, Li et al. 2011, 2016, Bober and Nair 2019), (ii) a redox route through a galactitol intermediate (using xylose reductase and xylitol dehydrogenase) (Liu et al. 2019), (iii) direct epimerization of D-fructose to tagatose using an engineered tagaturonate-3-epimerase (Shin et al. 2020), (iv) multistep conversion of fructose via phosphorylation, epimerization, and dephosphorylation (Lee et al. 2017), and finally, (v) conversion of glucose or disaccharides to tagatose via multistep isomerization and/or epimerization pathways (Dai et al. 2021a,b, Love et al. 2025, Palur et al. 2025). Through these works, researchers have found that conversions are often low at modest temperatures (<50°C) through direct *in vitro* isomerization (Love et al. 2025) and epimerization (Shin et al. 2020) pathways due to thermodynamics disfavoring tagatose formation. While increasing reaction temperature (to 70–90°C) (Kim et al. 2001) or addition of borate (Lim et al. 2007) has been shown to favor the equilibrium towards tagatose formation, there are significant drawbacks to each of these approaches—increased operating costs due to heating/cooling needs or additional purification requirements. Further, thermophilic enzymes often require supplementation of cations (Lee et al. 2005, Hong et al. 2011), an added expense, and high temperatures can degrade tagatose. The redox pathway through a galactitol intermediate can overcome the thermodynamic constraint at lower temperatures but requires the use of a sacrificial substrate to regenerate nicotinamide cofactors and drive the reaction forward (Liu et al. 2019). The same is true for the fructose phosphorylation-dephosphorylation pathway with ATP (Lee et al. 2017). Thus, there is still a need to develop alternative approaches to tagatose biosynthesis.

Previously, we described a biocatalyst design that allows the galactose to be isomerized to tagatose at high yields and titers using recombinant *Lactiplantibacillus plantarum* WCFS1 (Bober and Nair 2019)—a well-established probiotic bacterium with potential applications in biomanufacturing, food fermentation, and biomedicine. Our approach combined overexpression of a high-activity LAI with transporter-mediated substrate/product partitioning across the cell membrane and a detergent to facilitate rapid substrate uptake to achieve > 80% conversion at modest temperatures (50°C). Whole cell-based approaches can often overcome many of the limitations of cell-free reactions to drive the reaction forward, such as cofactor regeneration and differential compartmentalization of substrate and product (Kim et al. 2008, Bober and Nair 2019). However, one of the drawbacks of our prior approach was that the reaction proceeded rapidly to ~60% conversion in 6 h and then took another 42 h for the final ~30% conversion to > 80%. We reasoned that if *L. plantarum* could be engineered to be more thermotolerant, improved cellular and enzyme robustness may enable higher equilibrium conversion for the galactose to tagatose reaction.

Adaptive laboratory evolution (ALE) has emerged as a powerful engineering technique for bioprocess development, enabling engineering of novel cellular phenotypes through evolutionary selection processes (Sandberg et al. 2019, Lee and Kim 2020, Wang et al. 2023). The strength of ALE is that it enables engineering of complex traits where specific targets for rational engineering are not identifiable, where there may be the need to concurrently modify multiple cellular processes, or where directed genetic modifications are not possible or desirable. ALE has been widely and successfully applied to engineer strains with properties such as enhanced tolerance to growth inhibitors (Shui et al. 2015, Hemansi et al. 2022, Hartono et al. 2023, Lechtenberg et al. 2024, Yao et al. 2024, Chen et al. 2025), higher productivity (Mundhada et al. 2017, Wang et al. 2019, 2021, Yu et al. 2021, Jia et al. 2023), and thermal tolerance (Rudolph et al. 2010, Caspeta and Nielsen 2015, Chen et al. 2015, Huang et al. 2018, Dorau et al. 2021, García-Ríos et al. 2021, Li et al. 2024, Rychel et al. 2025, Yeh et al. 2025, Pattanakittivorakul et al. 2025, Pérez-Morales et al. 2025, 2026), etc. It has also been successfully used in combination with reverse genetics and/or systems biology approaches to elucidate novel genetic interactions or genotype-to-phenotype linkages (Anand et al. 2019, Phaneuf et al. 2020, Çetin et al. 2022, Gassler et al. 2022, Ingelman et al. 2025, Zhang et al. 2025, 2026).

In this work, we used ALE to thermally adapt *L. plantarum* to grow at elevated temperatures over 85 serial passages. We identified mutants that can grow at 43°C while maintaining their ability to grow at 37°C. We then attempted a galactose-to-tagatose bioconversion using this strain but found no improvement relative to the parental strain. Although our results did not advance tagatose biocatalysis, our work demonstrates successful engineering of an *L. plantarum* lineage capable of reproducible growth at elevated temperatures.

## Materials and methods

### General microbiology & molecular biology techniques

Growth of *Lactiplantibacillus plantarum* WCFS1 was evaluated across a temperature range of 37–52°C (at 3°C intervals) in MRS (de Man, Rogosa, Sharpe) broth. Overnight cultures grown for ~24 h at 37°C were used to inoculate pre-warmed medium at the indicated temperatures and adjusted to a uniform starting optical density (OD_600_). Cultures were incubated statically in a thermocycler, and growth was assessed by measuring OD_600_ at saturation.

### Tagatose production

*L. plantarum* cells transformed with LAI expression constructs (Bober and Nair 2019) were grown in MRS medium with 5 µg·mL^−1^ at 37°C overnight (~24 h). After 1:50 dilution, cells were grown at 37°C for 2.5 h and then induced with IP-673 (synthesized by Life Technologies; Carlsbad, CA) at a final concentration of 25 ng·mL^−1^ and 1 mM MgCl_2_ for 8 h before harvesting the cells by centrifugation at 3000 × g for 5 min. Cells were harvested via centrifugation and washed twice in phosphate-buffered saline pH 7.4 with 1.0 mM MgCl_2_ (PBSM). The enzyme activity assay was performed in 5 mL as follows: cell suspensions adjusted to an OD_600_ of 40 in PBSM and 300 mM D-galactose. Tagatose concentration in sterile-filtered supernatant was measured colorimetrically using the cysteine-carbazole-sulfuric-acid method with absorbance at 560 nm (Dische and Borenfreund 1951).

### Adaptive laboratory evolution (ALE) for thermal adaptation

Adaptive laboratory evolution (ALE) was performed to obtain *L. plantarum* WCFS1 derivatives with improved growth at elevated temperature. The parental strain was initially propagated in MRS broth at 37°C under aerobic conditions. The ALE experiment was initiated by inoculating a starter culture grown at 37°C into fresh 5 mL MRS broth and incubating under the same conditions. Subsequent evolution was carried out through serial batch passaging, with cultures diluted 1:10 into fresh pre-warmed 5 mL MRS medium at each transfer. Growth was monitored by measuring OD_600_ at 24, 48, and 72 h post-inoculation for every passage. Following initial growth at 37°C (passage #0), ALE was started by shifting cultures to 40°C. Cultures were incubated at this elevated temperature and assessed for growth across the 24–72 h time course. A passage was considered successful when reproducible growth (increase in OD_600_ relative to baseline) was observed within this time window. In cases where no growth occurred at the elevated temperature, the lineage was rescued by inoculating from the most recent adapted passage maintained at 37°C. Lineages needed to be rescued from 37°C cultures during passages #0, #3, #5, #10, #12, #13, #19, #23, #25, #54, #60, #85, and #87. The evolution experiment proceeded through multiple generations, with continued selection at elevated temperature until stable and reproducible growth was observed across ~2 consecutive passages. Evolution continued to passage #85, and populations were preserved as glycerol stocks at passages #15, #36, #52, and #85.

### Growth characterization of evolved populations

Evolved populations from selected passages were revived from glycerol stocks and evaluated for growth at 37°C and 43°C. Cultures grown for ~24 h were prepared at 37°C, normalized to a uniform starting OD_600_, and inoculated into fresh MRS medium pre-warmed to the test temperature. Growth assays were conducted in 10 mL deep-well plates and incubated at the indicated temperatures. OD_600_ was measured at defined time points to generate growth curves. For viable count analysis, cultures were plated onto pre-warmed MRS agar and incubated at 43°C, with parallel plating at 37°C as a control. Colonies were enumerated after incubation, and representative plates were imaged under identical conditions.

### Phenotypic screening across passages

To assess adaptation dynamics, isolates from different passages were screened for growth across 37–43°C. Colonies from archived stocks were grown overnight (~24 h) at 37°C and used to inoculate fresh medium at an initial OD_600_ of 0.05. Cultures were incubated at the indicated temperatures, and growth was measured after 24 h at 37°C and after 48 h at higher temperatures. Growth values from individual isolates were used to assess phenotypic variation across passages.

## Results and discussion

### Higher temperatures can promote tagatose formation

In prior work, we used the highly active L-arabinose isomerase (LAI) from *Lactobacillus sakei* (LsLAI) since it had been characterized as a highly active enzyme with an optimal reaction temperature of 37°C (Rhimi et al. 2010). However, the endogenous LAI from *L. plantarum* (LpLAI) has an optimal reaction temperature (50 − 60°C), making it a better choice for elevated temperature processes (Chouayekh et al. 2007, Zhang et al. 2007). We cloned both genes directly from their respective genomes and overexpressed them in *L. plantarum* using the high-copy p256 plasmid and the strong, inducible P_sppIP_ (a.k.a. P_orfX_) promoter, as described previously (Bober and Nair 2019). We assessed tagatose conversion with both enzymes using stationary phase cells (final OD_600_ = 40) incubated with 300 mM galactose for 3 h at 37, 50, and 60°C. Results indicated LsLAI yielded a monotonic increase in conversion with increasing temperature, whereas LpLAI had a maximum conversion at 50°C (Fig. 1a). This may be due to higher specific activity and/or stability of LsLAI, enabling high conversion at a wider temperature range. However, at the optimal temperature for LpLAI (50°C), it outperformed LsLAI. Thus, for processes operating at ~50°C, LpLAI may be the preferred enzyme. For processes below and above that narrow range, LsLAI may be the better choice. Due to the broader activity range, we decided to pursue work only with LsLAI.

**Figure 1:**
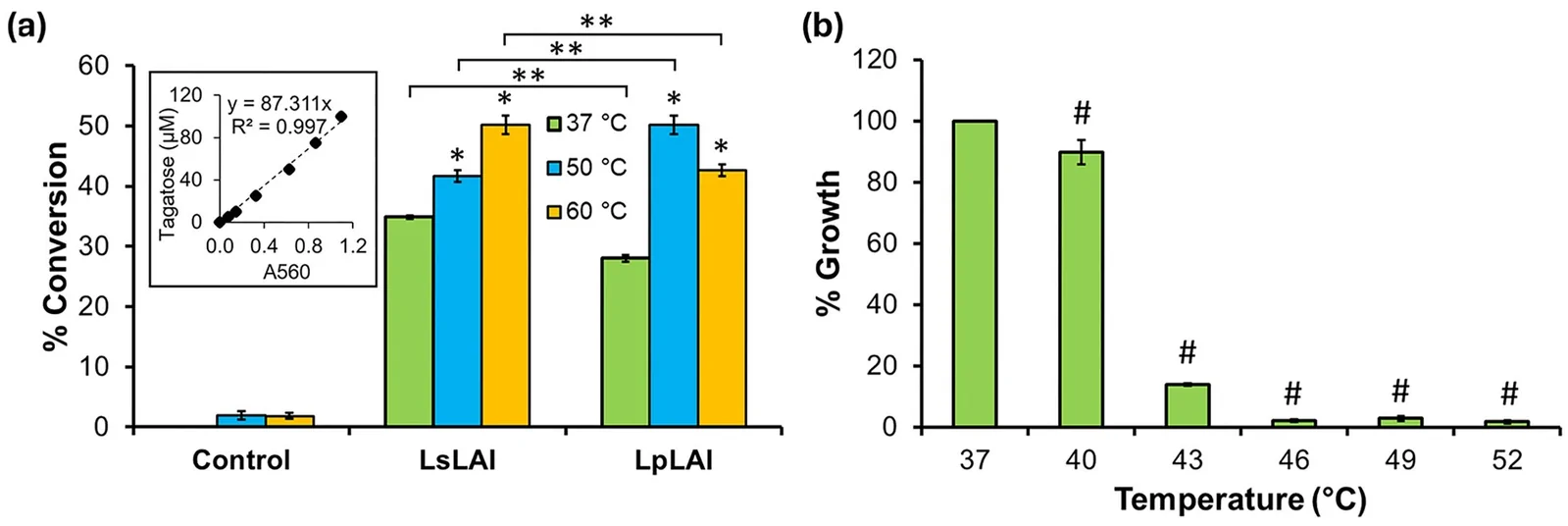
Temperature-dependence of tagatose production and growth. (a) Conversion of 300 mM galactose to tagatose with LsLAI- and LpLAI-expressing in high-density < I > L. plantarum</I > suspensions at varying temperatures. Parental (WT) <I > L. plantarum</I > was used as control. Inset: Calibration curve of the colorimetric cysteine-carbazole sulfuric acid assay used for tagatose quantification. * is *P* < 0.0001 vs. 37°C by 2-way ANOVA. * is *P* < 0.0001 between enzymes by 2-way ANOVA. (b) Growth of < I > L. plantarum</I > at different temperatures shows minimal growth at temperatures > 40°C. # is *P* < 0.001 vs. 37°C paired *t*-test. Data represents mean and standard deviation of 3 biological replicates.

### *Lactiplantibacillus plantarum* WCFS1 is modestly thermotolerant

Before initiating ALE, we assessed the tolerance of *L. plantarum* to different growth temperatures. Using cultures grown for ~24 h at 37°C, we inoculated parallel subcultures at temperatures between 37 and 52°C, in 3°C increments. At the end of 24 h, we measured the final OD_600_ of cells and normalized it to that of the 37°C culture. Results indicate that cells were able to grow well at 40°C but had a significant growth defect at temperatures ≥ 43°C (Fig. 1b). These results are consistent with prior publications where the authors observed a sharp decline in specific growth rate of *L. plantarum* from ~0.75 h^–1^ at 37°C to ~0.43 h^–1^ at 38°C (Matejčeková et al. 2016). When we attempted to regrow cultures at temperatures ≥ 40°C using serial passaging, cells failed to grow, indicating that *L. plantarum* can tolerate only a few doublings at elevated temperatures. Thus, *L. plantarum* is only modestly thermotolerant, especially compared to the model mesophilic bacterium, *E. coli*, which has been documented to grow well at 43°C and sometimes even at 44°C (Ron and Davis 1971). Thus, for our ALE studies with *L. plantarum*, we started by adapting cells for repeated growth at 40°C.

### *L. plantarum* populations can adapt for growth at higher temperatures

Previous studies have shown that ALE can be used to adapt bacteria (Rudolph et al. 2010, Chen et al. 2015, Dorau et al. 2021, Li et al. 2024, Pattanakittivorakul et al. 2025, Pérez-Morales et al. 2025, Rychel et al. 2025) and yeast (Caspeta and Nielsen 2015, Huang et al. 2018, García-Ríos et al. 2021) for growth at elevated temperatures, including the lactic acid bacterium, *Lactococcus lactis* (Chen et al. 2015, Dorau et al. 2021). However, as far as we know, *L. plantarum* has not evolved to grow at temperatures > 40°C. While our results show that *L. plantarum* was able to grow at 40°C, subsequent subcultures at this elevated temperature resulted in almost no growth. This suggests that while *L. plantarum* can double a few times at 40°C but cannot regrow once it transitions to the stationary phase and cannot be revived when maintained at this elevated temperature. Thus, we first adapted *L. plantarum* for reproducible growth at 40°C. Even after several attempts to maintain *L. plantarum* at 40°C for several cultures, we were unable to isolate cultures that could grow reproducibly at this temperature for multiple passages. To resolve this issue, we developed a cycling ALE protocol (Fig. 2a). After growing cultures at 40°C to saturation, we used them to inoculate parallel cultures at 40°C and 37°C. If the second culture at 40°C did not grow, we used the 37°C culture to start a new lineage at 40°C. This cycling between permissive and selective temperatures allowed cells to eventually evolve and adapt to growth at 40°C by passage #32, showing reproducible growth at the elevated temperatures over several subcultures.

**Figure 2:**
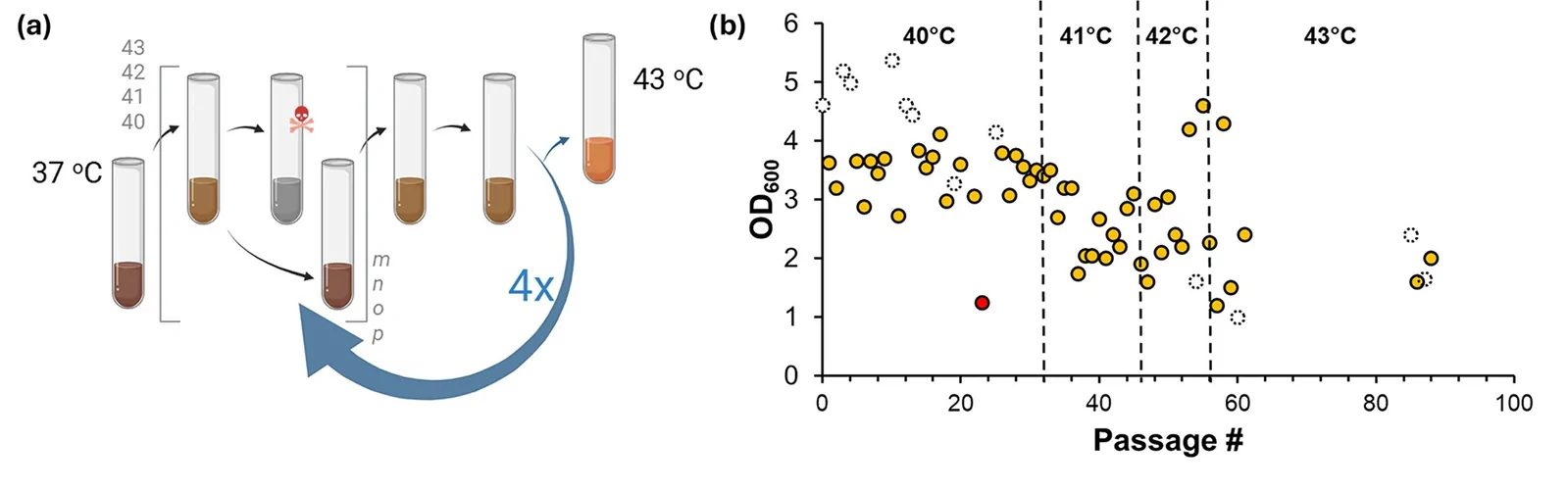
ALE of < I > L. plantarum</I > for growth at higher temperatures. (a) Schematic of ALE approach. Cultures grown at an elevated temperature (e.g., 40°C) were also passaged at 37°C, in parallel. Cultures that were able to grow over ≥ 2 passages at the elevated temperature were considered adapted and subsequently grown at an even higher temperature. If cultures failed to grow for ≥ 2 passages at the elevated temperature, the 37°C parallel culture was used to start a new lineage. This lineage restart needed to be done a different number of times for different temperature adaptations (40, 41, 42, 43°C). (b) End-point OD < sub > 600</sub > of ALE passages. Dotted circles are parallel passages at 37°C that were used to start a new lineage when temperature adaptation was incomplete. OD < sub > 600</sub > measurements were not taken for passages #62–#84. The red circle represents a culture that could not be revived at any temperature, and a previous passage needed to be used to continue the ALE.

We then increased the selective pressure to 41°C and then 42°C, continuing the parallel cultures at 37°C. Adaptation to 41°C was completed by passage #46 and to 42°C by passage #56. However, during this period, the final OD_600_ was significantly lower than that of cultures grown at 37°C. Fortunately, starting at passage #53, we found that OD_600_ started to rise again, which coincided with complete adaptation to 42°C. We started the ALE at 43°C and, after passage #85, found that the cells could be subcultured several times, indicating successful adaptation. The final OD_600_ for ALE cultures is shown in Fig. 2b. Final OD_600_ of 37°C cultures used to restart lineages (whenever measured) are shown as dotted circles. Due to technical limitations and restrictions during the COVID-19 pandemic, we were unable to measure OD_600_ for samples between passage #62 and #84 and several of the samples cultured at 37°C during adaptation studies.

To validate the evolutionary progression, we compared the growth of individual pools from across the different passages. We revived the pools from frozen stocks at 37°C and then inoculated them in parallel cultures at 37 and 43°C. Cultures from all passages grew at 37°C, albeit with different lengths of lag time (Fig. 3a). The parental strain grew the fastest, and the two early evolved passages—#15 and #36–showed an increase in lag phase from ~12 h to ~19 h. Thereafter, the lag times started decreasing and reduced to ~12 h and < 10 h for later passages #52 and #85. When comparing growth at 43°C, we found that only passages #52 and #85 showed any detectable growth over 60 h (Fig. 3b). Further, there was a marked decrease in lag time between passage #52 (~36 h) to ~20 h for passage #85. The longer lag times in these cultures could be due to the need to adapt from the pre-culture, which was grown at 37°C. Since only a single replicate was performed, assertions regarding lag time should not be taken to be conclusive. Regardless, these results confirm that the ALE and cycling strategy enabled evolution of populations capable of growing at 43°C while still maintaining growth at 37°C.

**Figure 3:**
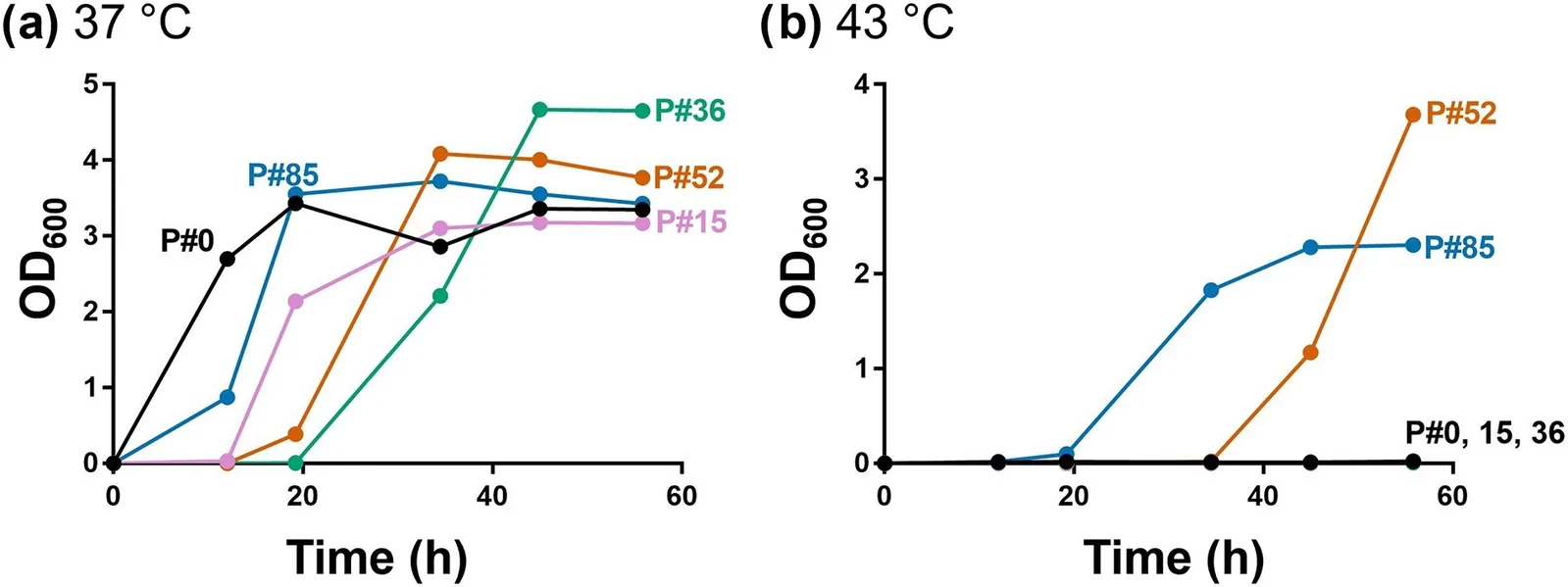
Growth comparison of evolved cultures. When growing (a) at 37°C, ALE resulted in an increase (#0→#15→#36) and then decrease (#36→#52→#85) in lag. (b) At 43°C, subsequent passages show shorter lag time between passages (#52→#85). Earlier passages (#0, #15, #36) failed to grow. Data represents a single replicate. P = passage.

### Individual strains are capable of growth at 43°C

Having confirmed that the ALE yielded populations with reproducible growth at 43°C, we wanted to ensure that individual isolates recapitulate the phenotypes of their respective pools. We plated 10^2^ − and 10^4^ − fold dilutions of 37°C precultures on MRS plates and incubated them at 37 and 43°C, respectively. A qualitative comparison agrees with Fig. 3, in that earlier passages (#0–#36, Fig. 4a–c) are unable to grow, whereas later passages (#52, #85, Fig. 4d–e) form distinct colonies at 43°C. We picked individual colonies from each passage grown at 37°C and grew them in a microtiter plate format at 37, 40, 43°C to saturation. As expected, earlier passages showed higher final ODs at 37°C (Fig. 5a), whereas subsequent passages showed higher ODs at elevated temperatures (Fig. 5b–c). These data confirm that not only do pooled cultures show enhanced tolerance to higher temperatures, but individual isolates do as well. Finally, to test the reproducibility of passage #85 isolates, we performed parallel cultures at 43°C (Fig. 5d). The absolute-agreement intraclass correlation coefficient was relatively low (ICC(A,1) = 0.283, bootstrap 95% CI 0.159–0.389), indicating limited agreement between the parallel cultures. Nevertheless, there was a moderate correlation between the two subcultures (Pearson’s r = 0.504, 95% CI 0.335–0.641).

**Figure 4:**
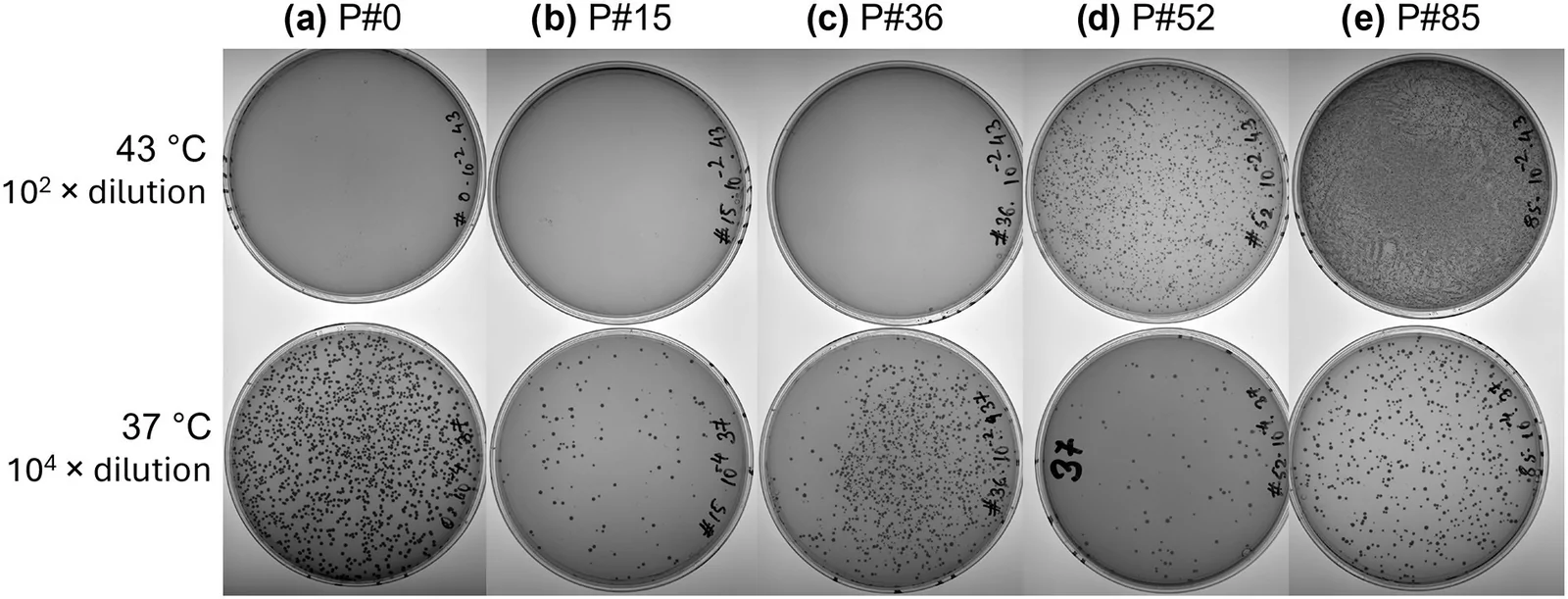
Qualitative comparison of thermal adaptation of individual pools & clones. Plating of cells from different passages (a) #0, (b) #15, (c) #36, (d) #52, (e) #85 at 37°C (10 < sup > 4</sup> × dilution) and 43°C (10 < sup > 2</sup> × dilution) shows that subsequent passages are adapted to grow at higher temperatures. Consistent with pooled culture data in Fig. 3, passages up to 36 cannot grow at 43°C. The final two passages show growth at 43°C, with #85 showing the largest number of colonies.

**Figure 5:**
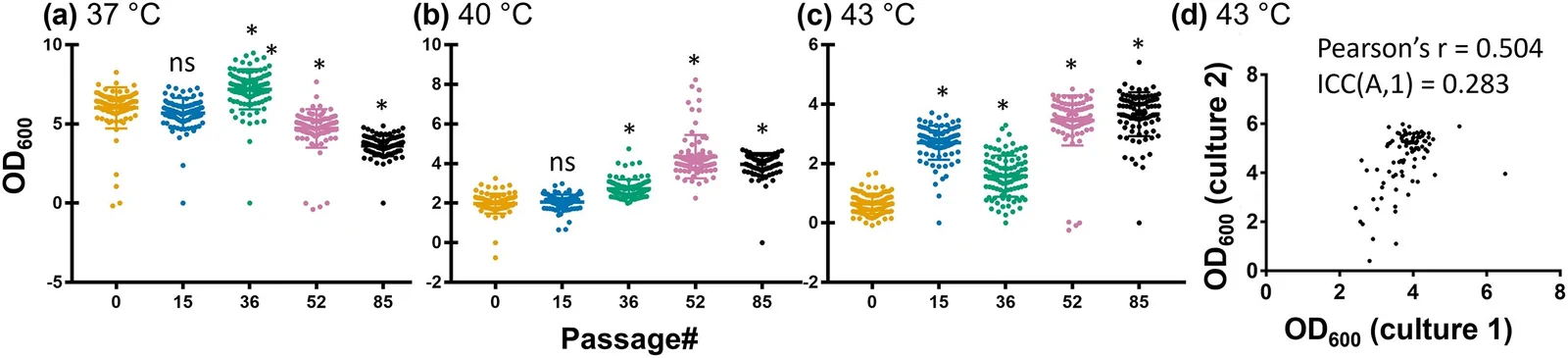
Quantitative comparison of thermal adaptation of passages. Clones from different passages were grown in liquid culture at (a) 37°C, (b) 40°C, and (c) 43°C. Their final OD < sub > 600</sub > was measured at saturation. Thermally adapted cells in passage #85 generally demonstrate lower growth at 37°C. * is *P* < 0.0001 vs. 37°C by 1-way ANOVA. Data represent 92 individual samples for each condition. (d) Growth of individual clones picked from passage #85 at 43°C shows poor reproducibility by individual clones (ICC(A,1) = 0.283, bootstrap 95% CI 0.159–0.389) but modest linear correlation between the two cultures (Pearson’s r = 0.504, 95% CI 0.335–0.641).

Previous studies have shown that thermal adaptation occurs due to a variety of mechanisms. In an early example with *Lactococcus cremoris* MG1363, the loss-of-function mutations in *gdpP* resulted in increased thermal tolerance. The gene encodes a membrane-bound cyclic-di-AMP phosphodiesterase and is involved in the signaling pathway for stress response (Smith et al. 2012). However, a later study did not report changes in *gdpP* in their ALE studies with the same bacterium but instead found mutations that upregulated *groESL* (chaperones) and *ribU* (riboflavin transporter) (Chen et al. 2015). In addition, the authors found mutations in *cdsA* (CDP diglyceride synthetase), involved in cell membrane metabolism, and *rpoC* (RNA polymerase β’ subunit), which led to large transcriptional changes. In the closely related *Lactococcus lactis* ATCC11454, temperature-adapted variants had mutations in *jag*, an RNA-binding protein implicated in cell division, *dnaB*, the replicative helicase, *argR*, a regulator of arginine metabolism, and *il_1403*, a cation (likely Zn^2+^) transporter (Papiran and Hamedi 2021). And finally, in *L. lactis* subsp. *lactis* biovar diacetylactis SD96, researchers attributed several changes leading to temperature adaptation, including peptidoglycan metabolism (*murC*), stringent response (*relA*), amino acid and nucleotide metabolism, and cell growth (*pheT, purR, rexA, dnaG, codY, ftsH*) (Dorau et al. 2021). Studies with other LAB have also found similar trends. In their study, Spus et al. found that heat-tolerance variants of *Lactobacillus helveticus* DSM 20075 had a mutation in Mn^2+^/Zn^2+^ cation transporter and several involved in glycerol metabolism (Spus et al. 2017). They implied that the latter mutations likely led to changes in cell membrane composition, given the importance of glycerol-3-phosphate in membrane synthesis. Min et al. found that thermal evolution *of Streptococcus thermophilus* BIOPOP-1 and BIOPOP-2 led to an increase in saturated fatty acid composition of the cell membrane (Min et al. 2020), and Jeon et al. found that mutations in MurD, involved in peptidoglycan metabolism, led to thermal resistance in *Lactobacillus acidophilus* EG004 (Jeon et al. 2021).

Based on the relatively consistent revelations from prior studies, it is likely that in evolved strains in passage #85, mutations are likely associated with stringent response, membrane/cell wall integrity, and/or chaperone upregulation. Future sequencing studies and genetic analyses of evolved mutants may help reveal the exact mechanism of adaptation.

### A thermally-adapted strain does not enhance tagatose biosynthesis

We picked a single isolate from passage #85 that grew reproducibly at 43°C ─ named “F12” ─ and assessed its suitability for whole-cell galactose-to-tagatose isomerization. Since galactose-to-tagatose isomerization productivity is enhanced at higher cell densities, experiments were conducted in 2 phases—the first for cell growth and protein expression and the second for tagatose production—as described previously (Bober and Nair 2019). After washing and concentration, we incubated them at different temperatures (37, 50, 60°C) to assess tagatose conversion. No statistically significant differences were observed between WT and F12 under the tested conditions (Fig. 6).

**Figure 6:**
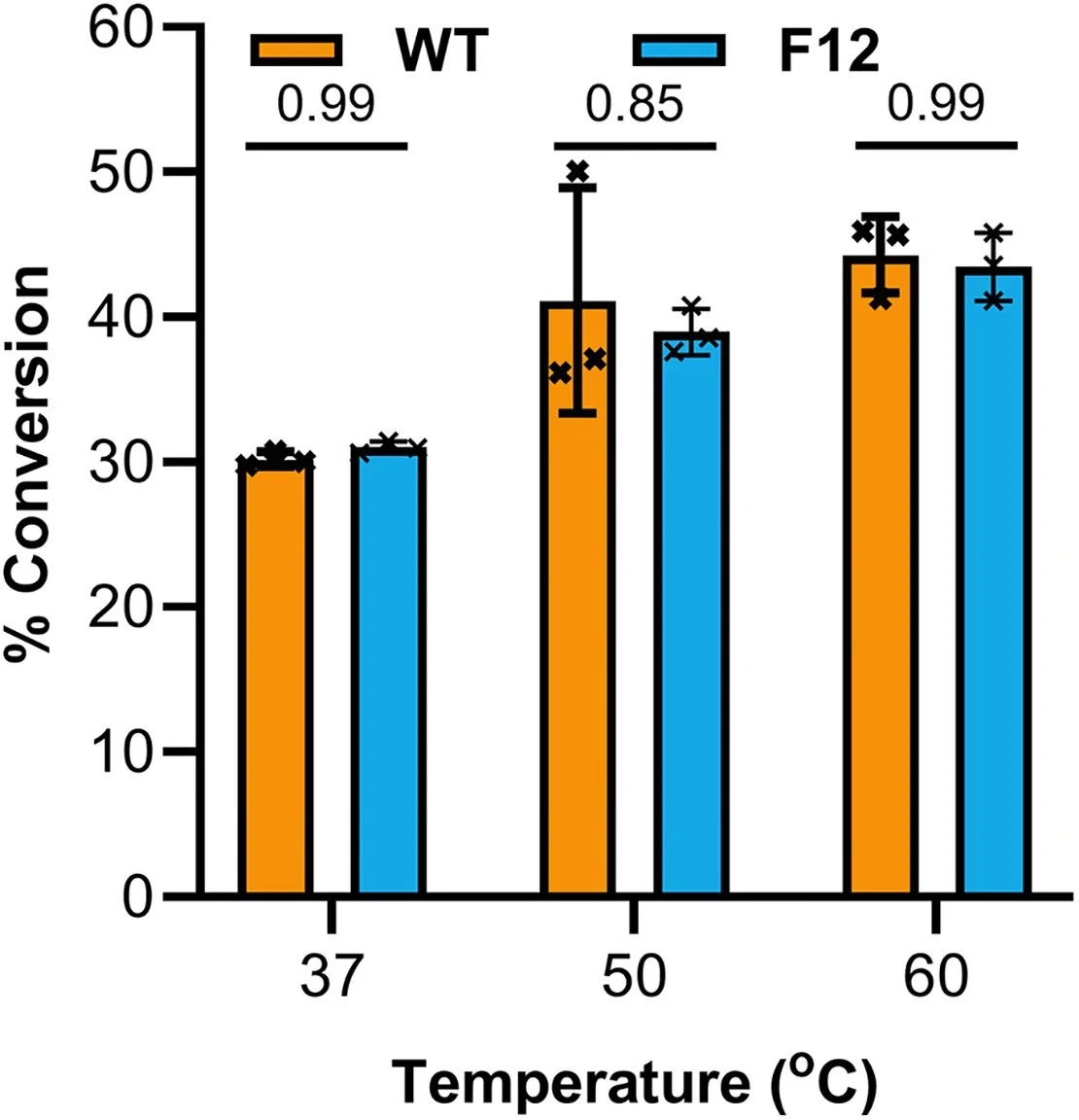
Galactose-to-tagatose isomerization in evolved strain. Effect of temperature on bioconversion by WT and F12 at 37, 50, and 60°C. Individual points represent independent biological replicate measurements (n = 3), and bars represent mean ± standard deviation (SD). Statistical significance was assessed using an ordinary two-way ANOVA with temperature and strain as factors, including their interaction. Šídák-adjusted p-values were 0.99, 0.85, and 0.99 at 37, 50, and 60°C, respectively, indicating no statistically significant differences.

There are likely several reasons why thermal adaptation did not increase the tagatose production in our evolved strain. Conversion in whole-cell tagatose biosynthesis is largely controlled by transport of galactose into and tagatose out of the cell (Bober and Nair 2019). Given the mechanisms of thermal adaptation observed in the literature and discussed above, ALE may not have led to any alterations in the transporter-based partitioning. It also seems that if chaperone overexpression was a mechanism by which thermal adaptation evolved, it did not affect the activity of sugar transporters meaningfully. Alternatively, altered membrane or cell wall rigidity may be counteracting any of the potential other benefits of the chaperone or transporter expression/folding.

## Conclusions

*Lactiplantibacillus plantarum* is a well-recognized probiotic with various potential applications in biomanufacturing, food fermentation, and biomedicine. In this study, we performed ALE to enhance the growth of *L. plantarum* in WCFS1 at 43°C compared to the parental strain, which cannot grow for extended periods at 40°C. To achieve this, we used a temperature cycling approach where evolutionary dead-ends during ALE at elevated temperatures were recovered by growth in permissive 37°C. Although the evolved isolate, F12, was unable to improve galactose-to-tagatose conversion, further characterization of this strain, especially using genomic sequencing, transcriptomics, and classical genetics approaches, could provide novel insight into the mechanism of thermal adaptation in this lactic acid bacterium.

## Data Availability

The data underlying this article are available in the article.
